# Do Bariatric Surgeries Enhance Brown/Beige Adipose Tissue Thermogenesis?

**DOI:** 10.3389/fendo.2020.00275

**Published:** 2020-04-30

**Authors:** Mohammed K. Hankir, Florian Seyfried

**Affiliations:** ^1^Department of Experimental Surgery, University Hospital Wuerzburg, Wuerzburg, Germany; ^2^Department of General, Visceral, Vascular and Pediatric Surgery, University Hospital Wuerzburg, Wuerzburg, Germany

**Keywords:** Roux-en-Y gastric bypass, vertical sleeve gastrectomy, brown adipose tissue, beige adipose tissue, thermogenesis, obesity, uncoupling protein 1, molecular and thermal imaging

## Abstract

Bariatric surgeries induce marked and durable weight loss in individuals with morbid obesity through powerful effects on both food intake and energy expenditure. While alterations in gut-brain communication are increasingly implicated in the improved eating behavior following bariatric surgeries, less is known about the mechanistic basis for energy expenditure changes. Brown adipose tissue (BAT) and beige adipose tissue (BeAT) have emerged as major regulators of whole-body energy metabolism in humans as well as in rodents due to their ability to convert the chemical energy in circulating glucose and fatty acids into heat. In this Review, we critically discuss the steadily growing evidence from preclinical and clinical studies suggesting that Roux-en-Y gastric bypass (RYGB) and vertical sleeve gastrectomy (VSG), the two most commonly performed bariatric surgeries, enhance BAT/BeAT thermogenesis. We address the documented mechanisms, highlight study limitations and finish by outlining unanswered questions in the subject. Further understanding how and to what extent bariatric surgeries enhance BAT/BeAT thermogenesis may not only aid in the development of improved obesity pharmacotherapies that safely and optimally target both sides of the energy balance equation, but also in the development of novel hyperglycemia and/or hyperlipidemia pharmacotherapies.

## Introduction

The growing obesity pandemic is thought to primarily stem from the increased intake of processed, caloric-dense foods coupled with an overall less active way of life (1). Despite the major health complications, heightened mortality risk, and significant socioeconomic burden associated with obesity (2–4), currently available treatments are of generally limited efficacy with the exception of bariatric surgeries (5). Roux-en-Y gastric bypass (RYGB) and vertical sleeve gastrectomy (VSG) are the two most commonly opted for procedures in the clinic (6), and sustainably induce marked weight loss associated with a host of other health benefits (7, 8). However, these procedures are last-resort measures due to their irreversible nature, expense and potential for complications (9). It is therefore a pressing medical need to develop safer, noninvasive alternatives to bariatric surgeries with wider applicability, which will invariably require a deeper understanding of their mechanistic underpinnings.

Because of the way they alter gastrointestinal anatomy, RYGB and VSG were first mistakenly (and still commonly) assumed to physically limit energy intake solely through the malabsorption and/or volumetric restriction of ingested food (10). It is now clear; however, that RYGB and VSG both lower defended body fat mass through complex effects on physiology (11, 12). This, to a large extent in humans, engenders marked and lasting reductions in food intake driven by alterations in gut-brain communication (13, 14). On the other hand, numerous clinical studies indicate that RYGB and VSG paradoxically decrease resting energy expenditure within the first year after surgery (15), although when normalized to body weight or fat-free mass, this converts to an increase which scales with the weight loss that they achieve (16–18). Adding further to the uncertainty, the situation in preclinical models appears to be quite the opposite, since food intake suppression by both RYGB and VSG are transient (19), while for the former procedure, total energy expenditure increases when expressed in absolute terms (20–22), or when normalized to body weight (23–25). These considerations and caveats aside, RYGB has reproducibly been shown to enhance diet-induced thermogenesis across species (17, 18, 23, 26, 27). Furthermore, while only established so far in rodents (25, 28), both RYGB and VSG likely at least limit the decrease in total energy expenditure that usually accompanies weight loss in humans too (29), possibly playing a decisive role in the successful long-term maintenance of a negative energy balance post-operatively. Indeed, weight regain in patients 2 years following RYGB has been attributed to its diminished effects on energy expenditure (30).

Incentivized by the rediscovery of functional brown adipose tissue (BAT) by 18F-Flurodeoxyglucose positron emission tomography-computed tomography (^18^F-FDG PET-CT) imaging in adult humans (31–37), surgical scientists wasted little time in addressing whether enhanced BAT thermogenesis may be responsible for the influences on total energy expenditure produced by RYGB and VSG described above. Similarly, the increased realization that energy-storing white adipose tissue (WAT) can adopt BAT-like characteristics by transforming into beige adipose tissue (BeAT) [also referred to as WAT “browning” (38)], has kindled interest in how RYGB and VSG may affect BeAT thermogenesis as well. In this Review, we will first provide a brief background to BAT and BeAT. We will then discuss, in chronological order, the steadily growing number of preclinical and clinical studies on the effects of RYGB and VSG on BAT and BeAT with particular emphasis on their methodology, limitations, mechanistic insights, and implications. We will finally highlight outstanding questions and present future perspectives.

## Bat And Beat

Because of the high energy demands and circulating nutrient uptake by BAT, ^18^F-FDG PET-CT imaging reveals the main classical depot in rodents and adult humans to be located in the interscapular region and the supraclavicular region, respectively (39). Physiologically, BAT is activated by sympathetic nerves when ambient temperatures drop to below thermoneutrality (referred to as temperature-induced thermogenesis) and during feeding (referred to as diet-induced thermogenesis) as part of physiological defense mechanisms in place to protect against hypothermia and weight gain, respectively (40, 41). Both these forms of adaptive thermogenesis absolutely depend on mitochondrial uncoupling protein 1 (UCP1) (42–45), a symporter embedded in the inner mitochondrial membrane that exothermically dissipates the proton gradient generated between the intermembrane space and the mitochondrial matrix by oxidative phosphorylation (46). Brown adipocytes are in fact optimized to generate heat in this way because of their high mitochondrial and UCP1 content as well as their multilocular morphology, which allows free fatty acids released by adrenergic-mediated lipolysis to efficiently activate UCP1 (47).

The anti-obesity potential of BAT was realized early on when its pharmacologic activation by selective beta 3 adrenergic receptor agonists protected leptin-deficient *ob/ob* mice and leptin receptor deficient *fa/fa* rats as well as wild-type rats on a high-fat diet from weight gain (48, 49). Furthermore, the sufficiency of BAT activation/expansion in protecting *ob/ob* mice and wild-type mice on a high-fat diet from weight gain has also been established (50). However, while acutely activating BAT with the selective beta 3 adrenergic agonist mirabegron or cold exposure markedly increases energy expenditure in healthy humans (37, 51), chronically activating/expanding BAT through daily mirabegron treatment, cold exposure or capsinoid treatment does not sufficiently cause weight loss *per se* (51, 52), although mirabegron treatment does markedly improve various aspects of glyceamic control (52). These findings suggest that in the clinical setting, recruiting BAT thermogenesis may better serve as an adjunct to promote and/or maintain weight loss and/or to exert other metabolic benefits (51, 52).

The term BeAT was coined to reflect its intermediate nature between WAT and BAT (38). BeAT forms when cells similar in size and morphology (multilocular and mitochondria-rich) and function (highly nutrient-consuming and thermogenic) to brown adipocytes arise in various WAT depots, most readily in subcutaneous WAT (such as the inguinal depot in rodents or the abdominal depot in humans), but also in visceral WAT [such as the gonadal depot in rodents and the omentum in humans (38)]. Evidence suggests that this can occur either through trans-differentiation of existing white adipocytes (53–57), or commitment of dedicated precursor cells (53, 58–60). As for BAT thermogenesis, BeAT thermogenesis is physiologically triggered in response to cold exposure and feeding through sympathetic nerve activation (61, 62).

Despite total BeAT depots quantitatively contributing ~ 60% less than BAT to temperature-induced thermogenesis due to lower UCP1 protein content (63), chronic activation of inguinal or gonadal BeAT thermogenesis by a variety of pharmacologic and genetic manipulations are sufficient to cause weight loss or protect from weight gain in mice on a high-fat diet (64–66). However, as for BAT thermogenesis (52), chronic activation of abdominal subcutaneous BeAT thermogenesis by mirabegron treatment in prediabetic, obese humans does not lead to significant weight loss, but it does markedly improve glycaemic control (67).

## RYGB and Bat/Beat Thermogenesis

An effective obesity treatment would in principle ideally target both sides of the energy balance equation. Indeed, evidence from preclinical studies suggests that in addition to suppressing food intake, liraglutide, which is a stable analog of the gut hormone glucagon-like peptide 1 (GLP-1) and 1 out of 5 currently approved obesity pharmacotherapies by the FDA (68), increases energy expenditure through stimulatory effects on BAT and BeAT thermogenesis (69, 70). However, this does not appear to be the case in diabetic patients (71), which may in part explain why liraglutide achieves relatively modest weight loss compared to bariatric surgeries in the clinic (5–10 vs. 20–30% within the first year, respectively) (72, 73).

In the first study attempting to address whether bariatric surgeries enhance BAT thermogenesis, Hankir et al. (74) performed small-animal ^18^F-FDG PET-CT imaging on high-fat diet-induced obese RYGB-operated and sham-operated male Wistar rats following acute treatment with a selective beta-3 adrenergic receptor agonist. Unexpectedly, they found that BAT ^18^F-FDG uptake was similar between surgical groups, as was BAT *Ucp1* mRNA expression determined by Northern Blot analysis following the same thermogenic stimulus (74). These findings provided early evidence against the outright enhancement of BAT thermogenesis by RYGB, although ^18^F-FDG PET-CT imaging has more recently been shown not to reliably reflect UCP1 thermogenic function in BAT (75, 76). Moreover, no measurements were made of energy expenditure by indirect calorimetry or heat production in this particular study, precluding any definitive conclusions about the effects of RYGB on BAT thermogenesis (74).

In a subsequent more rigorous study using the same RYGB model as Hankir et al. (74) applied to high-fat diet-induced obese male Wistar rats, Abegg et al. (77) performed indirect calorimetry as well as core-body and BAT temperature measurements by radiotelemetry. Here, the authors also cleverly varied the ambient housing temperature from mild cold stress (22°C), typically used in most rodent studies, to thermoneutrality (32°C), with the expectation that any increase in oxygen consumption from enhanced temperature-induced BAT thermogenesis in RYGB-operated rats at 22°C would be abrogated at 32°C (77). In addition to an obese sham-operated group, they also incorporated a body weight-matched (BWM) sham-operated control group to assess the specific weight loss-independent effects of RYGB. Unexpectedly, but in line with the earlier findings of Hankir et al. (74), there were no differences in energy expenditure between obese sham-operated and RYGB-operated groups or BAT/core-body temperatures regardless of the ambient housing temperature. However, RYGB clearly prevented the marked drop in these measures that was found for the BWM sham-operated control group (77). These findings suggest that while RYGB may not enhance temperature-induced thermogenesis *per se*, it does prevent the marked decrease that typically occurs with weight loss. These findings are also in line with comparable indirect calorimetry experiments performed on high-fat diet-induced obese mice in which RYGB similarly prevented the decrease in energy expenditure that occurs in BWM sham-operated mice—both under mild cold stress conditions and thermoneutrality (25). Interestingly, RYGB-operated mice had almost a third higher energy expenditure compared to BWM sham-operated mice during the onset of the dark period when they normally eat, further suggesting that RYGB enhances diet-induced thermogenesis (25). One way this could be achieved is through enhanced post-prandial release of the BAT-stimulating gut hormone secretin (78). Indeed, RYGB has recently been shown to markedly enhance post-prandial release of secretin in obese patients at both 1 week and 3 months post-operatively (79), although how this influences BAT thermogenesis remains to be formally assessed.

Due to the lack of a clear stimulatory effect of RYGB on BAT function in the study of Hankir et al. (74), two independent studies published at about the same time then addressed the effects of RYGB on BeAT formation (80, 81). Again using high-fat diet-induced obese RYGB-operated and sham-operated male Wistar rats as well as a BWM sham-operated control group, Hankir et al. (80) measured the mRNA expression by RT-qPCR of various genes that are essential for the thermogenic program in adipocytes including those that encode the mitochondrial proteins UCP1 and Cidea (82), the nuclear receptor co-activator and regulator of mitochondrial biogenesis peroxisome proliferator associated receptor gamma co-activator-1 alpha (PGC1-alpha) (83) and PRD1-BF1-RIZ1 homologous domain containing 16 (Prdm16) (84), as well as BeAT-specific markers such as V-erbA-related protein (Ear2) and transmembrane protein 26 (Tmem26) (85) in inguinal WAT and gonadal WAT. This analysis somewhat disappointingly revealed that there were no differences between any of the groups studied (80), although administering a beta 3 adrenergic agonist may have unmasked BeAT formation (38). Notably, RYGB at least partially prevented the decrease in BAT *Ucp1* mRNA expression that was found in the BWM sham-operated group compared to the obese sham-operated group (80). However, it should stressed that *Ucp1* mRNA levels do not provide a reliable indicator of UCP1 thermogenic function (86, 87), and no oxygen consumption or heat production measurements were made in this particular study - again precluding any definitive conclusions.

In the other study assessing BeAT formation by RYGB, Neinast et al. (81) employed high-fat diet-induced obese female C57BL/6 mice, which is highly relevant since a vast majority of bariatric surgeries in the clinic are performed on women. It was unexpectedly found that RYGB-operated mice had increased *Ucp1* mRNA expression determined by RT-qPCR and UCP1 protein expression determined by representative immunohistochemistry in gonadal WAT as opposed to in inguinal WAT, compared to obese sham-operated and BWM sham-operated mice (81). Additionally, in line with the lack of a stimulatory effect of RYGB on BAT thermogenic markers in male rats described above, RYGB had no effect on BAT *Ucp1* mRNA and UCP1 protein expressions. In order to gain insight into the mechanisms behind increased gonadal BeAT formation after RYGB, the authors next turned their attention to natriuretic peptides and their receptors, which have established roles in stimulating BeAT thermogenesis (88). They found using RT-qPCR an increase in gonadal WAT mRNA expression of B-natriuretic peptide (*Nppb*) and natriuretic peptide receptor 2 (*Npr2*) in RYGB-operated mice compared to both obese and BWM sham-operated mice in association with an increase in mRNA expression of the beta-3 adrenergic receptor (*Adrb3*). Together, these data suggest that RYGB increases B-natriuretic peptide synthesis in gonadal WAT to cell-autonomously enhance natriuretic peptide receptor 2 signaling in association with increased sympathetic tone, thereby enhancing BeAT formation in a weight-loss independent manner (81). However, it should be kept in mind that the findings from this particular study were only observational in nature and no energy expenditure or heat production measurements were made. Future studies are required employing indirect calorimetry on mice lacking either B-natriuretic peptide or natriuretic peptide receptor 2 to establish if RYGB does indeed enhance BeAT thermogenesis through the aforementioned mechanism.

The enhanced BeAT formation by RYGB would receive further support in subsequent observational mouse studies. He et al. (89) demonstrated using RT-qPCR that *Ucp1* and *Prdm16* as well as thermogenic *Ucp3* (90) mRNA expressions are higher in both the subcutaneous WAT and gonadal WAT of RYGB-operated diet-induced obese male mice compared to obese sham-operated mice. Further, in a comprehensive tissue-wide translational study, Ben-Zvi et al. (91) demonstrated using RNA-sequencing that *Ucp1* and *Cidea* mRNA expressions are higher in the inguinal WAT of RYGB-operated high-fat diet-induced obese male C57BL/6 mice compared to BWM sham-operated mice, as was UCP1 protein expression determined by representative immunohistochemistry. Importantly, this was complemented with indirect calorimetry measurements which confirmed higher energy expenditure in RYGB-operated mice compared to BWM sham-operated mice (91). While the underlying causal mechanisms for enhanced BeAT thermogenesis after RYGB compared to chronic caloric restriction-induced weight loss were not established in this particular study, mRNA expression of the anti-inflammatory cytokine interleukin-33 (*Il33*) was markedly increased in the inguinal WAT of RYGB-operated mice (91). This is relevant since systemically administered IL-33 can potently induce iWAT browning through a complex localized immune cell response involving type 2 innate lymphoid cell-derived IL-13 and eosinophil-derived IL-4 converging on IL-4 receptors on beige adipocyte precursors to induce their differentiation (92). Furthermore, growth hormone receptor expression was markedly reduced for RYGB-operated mice compared to BWM sham-operated mice throughout different tissues (91), which in the hypothalamus has the remarkable effect of sufficiently preventing the drop in BAT thermogenesis that usually accompanies chronic caloric-restriction induced weight loss (93).

The findings discussed so far collectively suggest that RYGB enhances BeAT thermogenesis but not BAT thermogenesis. However, this has been challenged in the most recent study on the subject by Chen et al. (94). The authors performed small-animal 18F-FDG PET-CT imaging on high-fat diet-induced obese RYGB-operated and obese sham-operated male C57BL/6 mice following acute treatment with insulin. They found that BAT ^18^F-FDG uptake on this occasion was increased by RYGB, but this simply means that BAT insulin sensitivity, as opposed to its thermogenic function, is enhanced (94). Nevertheless, RYGB increased BAT *Ucp1* and *Prdm16* mRNA expressions determined by RT-qPCR in association with increased energy expenditure determined by indirect calorimetry (94).

As informative as preclinical studies are, the ultimate goal is to understand if RYGB enhances BAT/BeAT thermogenesis in humans. Unfortunately, the number of clinical studies addressing this question is limited, likely due to the difficulties associated with obtaining ethical approval and recruiting patients. In an important early clinical study by Rachid et al. (95) addressing whether RYGB enhances BAT metabolic function, 12 obese, non-diabetic patients underwent 18F-FDG PET-CT imaging following acute cold exposure at baseline and 8 months post-operatively. To further establish whether RYGB enhances BAT thermogenic markers, supraclavicular biopsies were also collected at these time-points for gene expression analysis by RT-qPCR. It was found that while cold-induced supraclavicular BAT 18F-FDG uptake was unchanged by RYGB, *UCP1* mRNA expression increased (95) although how this would affect energy expenditure or heat production was not established. These findings are nevertheless in line with those of Piquer-Garcia et al. (96), who showed using infrared thermal imaging in 15 obese patients (9 with type 2 diabetes) that acute temperature-induced thermogenesis is not changed at 6 months after RYGB.

Concerning BeAT formation by RYGB, the tissue-wide RNA-sequencing translational study described above by Ben-Zvi et al. (91) revealed no effect on abdominal subcutaneous *UCP1* mRNA expression or any other standard thermogenic marker, although this was at a very early 1 month time-point post-operatively. Interestingly, in a study performed on 23 obese women by de Oliviera et al. (97) at baseline and the later 6 month time-point after RYGB, abdominal subcutaneous *UCP2* mRNA and perilipin 1 (PLIN1) mRNA expressions determined by RT-qPCR independently positively predicted weight loss. However, this did not correlate with the weight-adjusted increase in energy expenditure caused by RYGB (97). These preliminary findings nevertheless suggest that in humans, RYGB may increase BeAT thermogenesis through a UCP1-independent mechanism involving PLIN1-mediated fatty acid transfer from lipid droplets to UCP2. Under conditions of oxidative stress, potentially due to extensive remodeling of adipose tissue following RYGB, these liberated fatty acids could be rapidly oxidized to lipid hydroperoxides which activate UCP2-mediated proton transport and heat production (98).

## VSG and Bat/Beat Thermogenesis

Unlike RYGB, VSG does not involve gastrointestinal reconfiguration so it can reasonably be assumed to have a different effect on BAT/BeAT thermogenesis. Indeed, while there is considerable overlap in terms of the physiological changes after both procedures (99), there are some noticeable differences such as their effects on the gut microbiota (100–105) which have reported roles in positively regulating BAT/BeAT thermogenesis (106–109). In the first study addressing whether VSG enhances BAT thermogenesis, Baraboi et al. (110) performed a comprehensive set of experiments on high-fat diet-induced obese VSG-operated and sham-operated as well as BWM sham-operated male Wistar rats including small-animal ^18^F-Fluoro-6-thiaheptadecanoic acid (^18^F-FTHA) and ^11^C-acetate PET-CT imaging, which measure fatty acid utilization and mitochondrial beta oxidation, respectively, in conjunction with indirect calorimetry measurements (110). Similar to RYGB in rats described by Abegg et al. (77), VSG did not increase total energy expenditure, but it did prevent the drop that occurred in BWM sham-operated rats compared to obese sham-operated rats (110). Additionally, similar to RYGB in rats described by Hankir et al. (80), VSG prevented the drop in BAT *Ucp1* mRNA expression determined by RT-qPCR that occurred in BWM sham-operated rats compared to obese sham-operated rats (110). Interestingly, VSG increased both BAT ^18^F-FTHA and ^11^C-acetate uptake, indicating increased brown adipocyte fatty acid utilization and beta-oxidation, respectively, compared to both obese sham-operated and BWM sham-operated rats (110). These findings suggest that while VSG may enhance BAT thermogenesis, it is only sufficient to prevent the decrease in total energy expenditure that usually accompanies caloric restriction-induced weight loss rather than enhancing it overall. This is nevertheless consistent with a subsequent study by Moncada et al. (111) on high-fat diet-induced obese VSG-operated and obese sham-operated as well as pair-fed (PF) sham-operated male Wistar rats that underwent core-body temperature measurements. It was found that VSG increased core-body temperature compared to obese sham-operated rats in association with increased BAT UCP1 protein levels determined by Western Blot (111). Interestingly, the increase in these measures for VSG-operated rats compared to PF sham-operated rats was only noticeable in those not characterized as obesity-prone, suggesting that there is a genetic component to the effectiveness of VSG in enhancing BAT thermogenesis (111).

Having established that VSG consistently enhances BAT thermogenesis in rats, efforts were then made to elucidate the underlying mechanisms. Because plasma bile acids are known to be markedly increased by VSG (112), and agonists of the bile acid receptor TGR5 promote BAT thermogenesis in rodents and humans (113, 114), their role in enhanced BAT thermogenesis after VSG was tested in male high-fat diet-induced obese mice (115). First, it was shown that TGR5 knockout mice regained weight after VSG, unlike wild-type mice, and that this was despite similar food intake between genotypes (115). Accordingly, TGR5 knockout mice did not exhibit the increase in energy expenditure determined by indirect calorimetry after VSG that occurs in wild-type mice (115). In line with these findings, the increase in BAT *Ucp1, Ucp3*, and *Pgc1alpha* mRNA expressions determined by RT-qPCR caused by VSG in wild-type mice was not seen in TGR5 knockout mice (115). These results strongly suggest that the increase in circulating bile acids caused by VSG leads to TGR5-mediated enhancement in BAT thermogenesis thereby contributing to a negative whole-body energy balance (115). However, this conclusion would be more strongly supported by performing VSG on mice lacking TGR5 specifically in BAT.

To evaluate if VSG enhances BeAT formation and assess in detail the underlying mechanisms, Liu et al. (116) employed streptozotocin-induced diabetic VSG-operated, obese sham-operated and FR sham-operated male Sprague Dawley rats. It was found that VSG increased *Ucp1* and *Pgc1alpha* mRNA expressions determined by RT-qPCR as well as UCP1 and PGC-1alpha protein levels determined by both Western Blot and representative immunohistochemistry in inguinal WAT (116). This was associated with a strong (20-fold) increase in sirtuin 1 (*Sirt1*) and adiponectin (*Adipoq*) mRNA expressions and an increase in Sirt1 and adiponectin protein levels in inguinal WAT. Mechanistic experiments in cultured 3T3-L1 cells, a mouse white adipocyte cell line, then revealed that forced overexpression of adiponectin increased Sirtuin 1, UCP1, and PGC-1alpha protein levels. In turn, forced overexpression of Sirt1 increased AMPK activation determined by phosphorylated threonine 172 immunoblotting. Because AMPK can increase the expression of PGC-1alpha and UCP1 in BeAT (117), its causal role in BeAT formation after VSG was tested (115). Strikingly, chronic (1 month) weekly administration of the AMPK inhibitor compound C prevented the increase in *Ucp1* and *Pgc1alpha* mRNA expressions as well as UCP1 and PGC1-alpha protein levels in inguinal WAT caused by VSG (115). Taken together, these impressive results suggest that VSG first increases adiponectin expression and release from WAT, which in turn cell-autonomously increases SIRT1 and AMPK activities to drive BeAT formation (116). It is unfortunate however that no measurements of energy expenditure were made by indirect calorimetry in this particular study. Moreover, it is unclear how inhibition of BeAT formation by compound C may have affected body weight or any other metabolic parameter in VSG-operated animals.

In a recent study by Harris et al. (118) aimed at elucidating the weight loss-independent mechanisms behind improved glycaemic control following VSG, interesting effects on adipose tissue metabolic function and gene expression pertinent to thermogenesis were made. Non-obese C57Bl/6 male mice maintained on a normal chow diet first underwent VSG or sham surgeries. Two weeks later, they were orally administered ^18^F-FDG and its tissue distribution after 1 h was quantified *ex vivo*. Remarkably, this revealed that VSG significantly increased ^18^F-FDG uptake in iWAT and eWAT compared to sham surgery despite similar body weights and energy expenditure determined by indirect calorimetry between groups. Additionally, VSG-operated mice exhibited improved oral glucose tolerance and increased systemic carbohydrate metabolism (118). Since ^18^F-FDG uptake by iWAT is increased by beta 3 adrenergic receptor agonist-induced and cold-induced browning (39), these findings provide further functional evidence of iWAT browning after VSG and novel evidence of eWAT browning and suggest that this contributes to improved glycaemic control but not to energy expenditure changes post-operatively. Interestingly, eWAT browning by VSG may be independent of sympathetic nervous system function, since neither chronic pharmacologic beta 3 adrenergic receptor activation nor cold exposure increases eWAT ^18^F-FDG uptake (39). The authors further went on to show through RNA-sequencing that VSG reduces eWAT mRNA levels of suppressor of zeste 12 protein homolog (*Suz12*) which forms part of the polycomb repressor complex 2 (PRC2) (119). This could potentially contribute to reduced methyltransferase activity of PRC2, leading to reduced lysine 27 trimethylation of histone H3 at the *Ucp1* and *Pgc1alpha* promoters, thereby disinhibiting their transcription (119). It would be interesting in future studies to perform similar experiments as those of Harris et al. (118) on mice at later timepoints after VSG to assess its long-term effects on whole-body energy and glucose balance in relation to brown and beige adipose tissue thermogenesis.

Lastly, to ascertain the effects of bariatric surgery (8 RYGB and 15 VSG) on BAT function in humans, Dadson et al. (120) performed ^18^F-FTHA PET-CT imaging on 23 obese patients (10 with type 2 diabetes) at baseline and 6 months post-operatively. It was found that bariatric surgery increased supraclavicular BAT ^18^F-FTHA uptake to the level of lean controls. Notably, BAT triglyceride content was reduced by bariatric surgery as revealed by CT, which is a proxy of enhanced BAT thermogenesis (121). While this did not correlate with post-operative changes in energy expenditure determined by indirect calorimetry, it positively correlated with post-operative improvements in insulin sensitivity (120). The findings again suggest that while VSG may enhance BAT thermogenesis, this does not increase energy expenditure overall but may contribute to improved glycaemic control. The enhanced BAT thermogenesis by VSG would receive direct support in a subsequent study by Picquer-Garcia et al. (96). They demonstrated using infrared thermal imaging on 15 obese patients (5 with type 2 diabetes) that VSG significantly increased acute temperature-induced thermogenesis at the 6 month post-operative time-point.

Concerning the effects of VSG on BeAT formation, Jahansouz et al. (122) collected abdominal subcutaneous adipose tissue samples from 20 obese patients at baseline and 1 week post-operatively (122). Surprisingly, UCP1 protein levels determined by Western Blot were reduced by VSG. In contrast, *UCP2* mRNA, and *PLIN2* mRNA expressions in abdominal subcutaneous adipose tissue determined by RT-qPCR were increased by VSG which associated with a 15-fold higher beta-oxidation rate *ex vivo* (122). These findings are in line with those of Tarabra et al. (123) who collected abdominal subcutaneous adipose tissue from 4 to 9 obese girls at baseline and 10 months after VSG. It was found that VSG markedly increased PLIN1 protein levels determined by Western Blot with no changes in *UCP1* mRNA levels determined by RT-qPCR. However, VSG increased mRNA expression of *PGC1alpha, CIDEA, TBX1, and ADIPOQ*. Interestingly, they found that omental adipose tissue had greater *UCP1, PGC1alpha*, and *CIDEA* mRNA expressions determined by RT-qPCR and UCP1 protein expression revealed by representative immunohistochemistry than abdominal subcutaneous adipose tissue at baseline in these patients, which may have further increased following VSG. Together, the findings of Jahansouz et al. (122) and Tarabra et al. (123) intriguingly suggest that as for RYGB in humans described earlier (97), VSG may enhance (abdominal subcutaneous) BeAT thermogenesis through a UCP1-independent, PLIN1/2- and UCP2-dependent mechanism under conditions of oxidative stress.

## Conclusions and Future Studies

**Figure 1:**
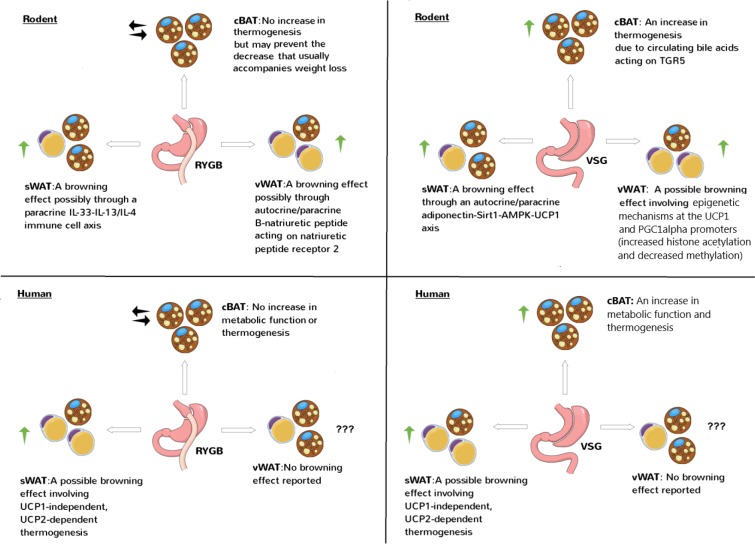
Effects of RYGB and VSG on brown and beige adipose tissue thermogenesis in rodents and humans. The studies discussed in this Review generally show a stimulatory effect of RYGB and VSG on brown and/or beige adipose tissue thermogenesis involving canonical, UCP1-dependent or non-canonical, UCP1-independent mechanisms. cBAT refers to classical BAT which is the interscapular depot in rodents and supraclavicular depot in humans. sWAT refers to subcutaneous WAT which is the inguinal depot in rodents and abdominal depot in humans. vWAT refers to visceral WAT which is the epigonadal depot in rodents and omental depot in humans. Note how sWAT in rodents and vWAT in humans have more browning potential, respectively.

The rodent and human studies discussed in this Review together suggest that RYGB mainly enhances BeAT thermogenesis while VSG mainly enhances BAT thermogenesis (Figure 1). Interestingly, in the rodent studies in which BAT/BeAT were not clearly recruited by RYGB (74, 77, 80), animals were post-operatively maintained on a low-fat diet unlike the high-fat diet used in other studies (81, 89, 91, 94). This suggests that high-fat diets are key determinants of BAT/BeAT thermogenesis post-operatively.

Apart for bile acids in the case for VSG, it is unclear which peripheral signals are causally required for enhanced BAT/BeAT thermogenesis after bariatric surgeries which could differ according to procedure. A role for GLP-1 and the co-released gut hormone peptide tyrosine tyrosine (PYY), the augmented post-prandrial levels of which are a hallmark of both RYGB and VSG (99), is at least unlikely for RYGB. This is because systemic and central administration of the GLP-1 receptor antagonist exendin-9 was shown not to influence total energy expenditure in RYGB-operated rats (124, 125) and mice with combined deficiency in the GLP-1 receptor and Y2 receptor remain fully protected by RYGB from the decrease in energy expenditure that usually accompanies caloric-restriction-induced weight loss (126). Postoperative shifts in the intestinal microbiota could also potentially be responsible, especially considering that their depletion by antibiotics prevents some of the metabolic benefits of VSG in diet-induced obese mice through effects on inguinal WAT (127). Moreover, the causal role of BAT/BeAT thermogenesis in weight loss after bariatric surgeries is still unclear. One way to formally address this issue is to perform VSG and RYGB on UCP1-deficient mice. In the event that these mice respond normally to bariatric surgeries, then a role for BAT/BeAT thermogenesis in causing weight loss post-operatively should not be fully discounted as UCP1-independent modes of thermogenesis are increasingly being described in BAT/BeAT (128). Indeed, a UCP1-independent form of thermogenesis involving AMPK in inguinal WAT has recently been shown (129), which may operate after VSG in particular (116).

The fact that increases in energy expenditure and BAT/BeAT thermogenesis seem to be weaker or even absent in humans compared to rodents and especially mice after RYGB and VSG (19), serves only to emphasize that animal models of bariatric surgeries can provide novel mechanisms for how to promote marked and lasting weight loss. Furthermore, a direct comparison between the effects of dieting-induced weight loss with RYGB and VSG on energy expenditure and BAT/BeAT thermogenesis still needs to be performed in humans, which may translate what has already been shown in rodents. Finally, it is possible that BAT/BeAT thermogenesis may play important roles beyond the body weight loss induced by bariatric surgeries, such as in improving glycaemic control and lipid homeostasis (52, 67, 130, 131).

## Author Contributions

MH wrote the manuscript and produced the figure. FS contributed to the manuscript.

## Conflict of Interest

The authors declare that the research was conducted in the absence of any commercial or financial relationships that could be construed as a potential conflict of interest.
